# Molecular surveillance and genetic diversity of *Klossiella equi* in European equids using kidney and urine samples

**DOI:** 10.3389/fvets.2026.1862055

**Published:** 2026-07-13

**Authors:** Sára Lukšíková, Ondřej Daněk, Francesco Buono, Stefan O. Rabei, Eduardo Berriatua, Pedro Sánchez, Štěpán Bodeček, Petr Jahn, Mariel G. Pikkemaat, Elizabeth G. Zeldenrust, Vincenzo Veneziano, Andrei D. Mihalca, David Modrý

**Affiliations:** 1 Department of Veterinary Sciences, Faculty of Agrobiology, Food, and Natural Resources, Czech University of Life Sciences, Prague, Czechia; 2 Department of Veterinary Medicine and Animal Production, University of Naples Federico II, Naples, Italy; 3 Department of Parasitology and Parasitic Diseases, University of Agricultural Sciences and Veterinary Medicine of Cluj-Napoca, Cluj-Napoca, Romania; 4 Animal Health Department, University of Murcia, Murcia, Spain; 5 Equine Clinic, Faculty of Veterinary Medicine, University of Veterinary Sciences Brno, Brno, Czechia; 6 Wageningen Food Safety Research (WFSR), Wageningen University & Research, Wageningen, Netherlands; 7 Department of Pathobiology, Ontario Veterinary College, University of Guelph, Guelph, ON, Canada; 8 Department of Botany and Zoology, Faculty of Science, Masaryk University, Brno, Czechia; 9 Biology Center, Institute of Parasitology, Czech Academy of Sciences, České Budějovice, Czechia

**Keywords:** Adeleorina, equids, kidney, *Klossiella equi*, parasite, urine

## Abstract

**Introduction:**

*Klossiella equi* is a renal apicomplexan parasite of equids, yet current knowledge regarding its clinical impact, geographic distribution and genetic diversity is limited.

**Methods:**

This study investigated apparent molecular prevalence and genetic variability of *K. equi* in opportunistically collected kidney and urine samples from 284 equids across five European countries (the Czech Republic, Italy, the Netherlands, Romania, and Spain). Detection was determined using a conventional PCR assay. Genetic analysis using mitochondrial markers was performed on a subset of 38 PCR-positive samples, selected to ensure geographic representation.

**Results:**

*Klossiella equi* DNA was detected in 33% of sampled equids (94/284) with positivity rates of 31.0% in kidney and 25.6% in urine. Agreement between 82 paired kidney-urine samples was moderate (Cohen’s kappa=0.5; 95% CI: 0.3–0.7). Using a composite reference standard, urine testing showed an apparent sensitivity and specificity of 65.6% and 100%, respectively. Sanger sequencing of the selected subset revealed low genetic variability with 2 (*cytB*) to 4 (*COI*) observed haplotypes per marker and limited divergence among European isolates. The most genetically distinct haplotypes for all three markers were detected in Italian mule and donkey samples.

**Discussion:**

These findings indicate that *K. equi* occurs in equids from multiple European countries and exhibits low genetic variability. Despite limited sensitivity, urine PCR may serve as non-invasive screening and surveillance tool for *K. equi* and should enable further research addressing epidemiology, potential pathogenicity and clinical relevance of this equine parasite.

## 1 Introduction

Equine veterinary parasitology is largely dominated by attention to helminth infections, which remain a primary focus of veterinary care and parasite control. Among the most common endoparasites of equids worldwide are small strongyles (1, 49). The negative impacts of intestinal nematodes (i.e., small strongyles and *Parascaris* spp.) and cestodes (*Anoplocephala perfoliata*), together with growing anthelmintic resistance are addressed by several recent studies (2–5).

In contrast, protozoan parasites are often overlooked in routine practice, despite their global distribution and potential to cause severe disease. Vector-borne protozoan pathogens like *Leishmania martiniquensis* and *Leishmania infantum* (both are causative agents of leishmaniasis), *Besnoitia bennetti* (the causative agent of besnoitiosis) and the causative agents of piroplasmosis, *Theileria equi* and *Babesia caballi*, are beginning to attract the attention of equine clinicians (6–11, 49). Monoxenous gastrointestinal apicomplexan parasites such as *Eimeria leuckarti* and *Cryptosporidium* species are comparatively understudied and remain in the shade of their vector-borne counterparts (12–14). To overcome limits of conventional microscopy-based diagnostic, the DNA-based approaches greatly expanded the spectrum of diagnostic tools available in veterinary parasitology, uncovering the distribution and diversity of several neglected pathogens including *Klossiella equi*, the renal apicomplexan parasite of equids.

The genus *Klossiella* was established upon the description of *Klossiella muris* by Smith and Johnson (15) in the renal epithelium of mice. The genus is characterized by a remarkable diversity of mammalian hosts. In addition to equids and mice, species have been confirmed in guinea pigs [*K. cobayae*, (16)], bats [*K. killicki*, (17)] and various marsupials, including sugar gliders [*K. dulcis* (18)], kangaroos [*K. rufi*, (19)], and bandicoots [*K. quimrensis* (20)].

*Klossiella equi* Bauman, 1946 is the only known apicomplexan adeleorinid protist that infects the renal parenchyma of horses (26), (21), donkeys (22) and zebras (23). The monoxenous life cycle is believed to begin with the ingestion of infective sporocysts via contaminated feed or water. Following ingestion, sporozoites are released in the gastrointestinal tract, migrate via bloodstream to the renal epithelium, where they undergo merogony, followed by gametogony and sporogony. This process culminates in the formation of thin-walled oocysts within the tubular cells. Oocysts rupture and sporocysts are then excreted via urine into the external environment (24). Like in other members of the genus *Klossiella*, merogony of *K. equi* takes place in the Bowman’s capsule and proximal tubule epithelium, whereas gametogony and sporogony are restricted to the thick limb of the Henle’s loop (21, 24).

Although infections with *K. equi* are typically considered asymptomatic, they can occasionally lead to significant renal complications, particularly in immunocompromised horses (25) and enhance the susceptibility of the kidneys to tubular injury (26). Heavy infections may induce severe lesions, including rupture of the renal tubules and lymphoplasmacytic interstitial nephritis (27). The parasite can also apparently induce detectable clinical abnormalities. Ballweber et al. (25) identified a direct association between *K. equi* infection and hematuria, and Baker et al. (26) provided notable documentation of clinical signs of acute kidney injury (AKI), including polyuria and polydipsia, in direct association with *K. equi* infection. Similarly, the intratubular stages of *K. muris* have been associated with multifocal mild tubular necrosis accompanied by focal interstitial infiltrates of lymphocytes and plasma cells in mice (50). *Klossiella equi* exhibits a near-global distribution, having been reported across all continents except Antarctica (21–23, 28, 29). Despite this wide geographical range, its occurrence in Europe remains largely unexplored, and its clinical significance is not well understood, partly due to the lack of reliable intravital diagnostic tools. The intravital diagnosis of *K. equi* is difficult considering that sporocysts in urine of infected equids are not easily concentrated (25); thus, prior to the DNA-based diagnostics, the detection of *K. equi* has relied primarily on postmortem tissue examination and histological detection of endogenous stages of the parasite within the kidneys (24, 30, 31).

To address these gaps, our study investigated the molecular detection frequency and genetic diversity of *K. equi* in an opportunistically collected sample set derived mainly from slaughterhouse and post-mortem material. Moreover, we evaluated the utility of urine-derived DNA detection as a non-invasive diagnostic alternative to post-mortem examinations.

## 2 Materials and methods

### 2.1 Sampling and study area

Between 2023 and 2025, 284 individual kidney samples were opportunistically collected during postmortem examination; 277 (97.5%) from horses, 6 (2.1%) from mules and 1 (0.4%) from a donkey across five European countries—Italy (*n* = 46; 16.2%), Netherlands (*n* = 100; 35.2%), Spain (*n* = 30; 10.6%), Romania (*n* = 96; 33.8%), and Czechia (*n* = 12; 4.2%). The majority of samples (267; 94.0%) were sourced from horses at slaughterhouses; exceptions included 5 horses (1.8%) from Italy (death due to colic) and 12 horses (4.2%) from Czechia (euthanized due to critical illness). In addition, 82 urine samples were collected from the bladder of some of these horses in Spain (*n* = 28; 34.1%), Romania (*n* = 51; 62.2%) and Czechia (*n* = 3; 3.7%).

**Figure 1:**
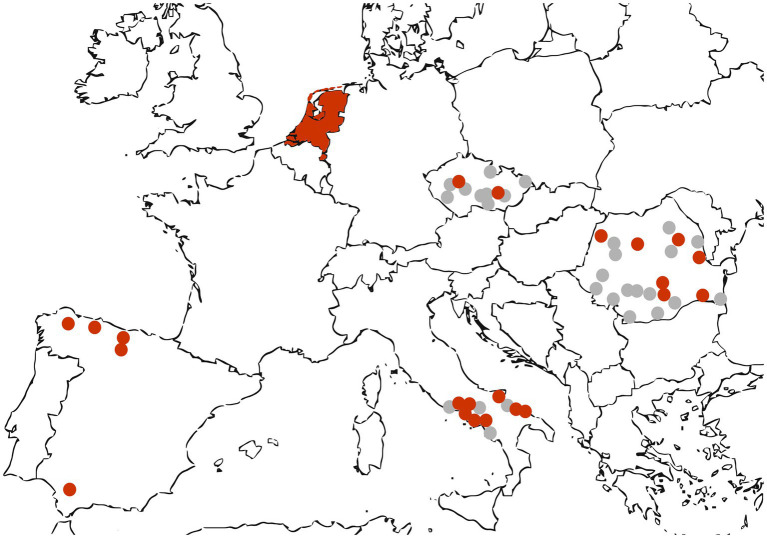
Map of Europe with schematized approximate locations in Czechia, Italy, Spain and Romania from which equine samples were obtained (gray spots—negative samples only, red spots—positive findings). Particular locations for each sample are listed in Supplementary Table 1. In the case of Netherlands exact location data were not available, thus the occurrence is represented for the whole country (see Materials and Methods). Source: world_borders.shp; The world_borders.zip dataset is Shapefile of the world’s international borders, derived by Schuyler Erle from public domain sources _ KoenB, Wikimedia Commons. Public domain.

In total, the study included 82 paired samples (matched kidney and urine), collected from the same individual horses for comparative molecular analysis. The origin of sampled equids is illustrated in Figure 1. In cases from Netherlands, location data were not available, thus the occurrence is represented for the entire country. In Spain, the equids were born and raised on pastures in northern regions (Cantabria, Asturias, Palencia and Galicia) until approximately 6 months of age, after which they were transferred to a feedlot in southern Spain (Seville) for fattening until 17–20 months old.

Collected kidney and urine samples were stored at −20 °C until they were processed for *K. equi* detection.

### 2.2 DNA extraction and PCR amplification

Following thawing, a piece of kidney tissue was excised from the cortico-medullary junction to ensure the inclusion of both cortical and medullary regions, maximizing the capture of relevant nephron segments, considering that the life cycle stages of *K. equi* occur within both the glomeruli and the loop of Henle (21). To eliminate the potential for DNA cross-contamination during tissue excision, stringent anti-contamination protocols were enforced throughout sampling. This involved utilizing a new, sterile surgical blade for every sample and exchanging gloves between the processing of individual kidneys. Furthermore, to prevent indirect contact contamination, multiple layers of disposable filtration paper were placed beneath each kidney and immediately discarded post-sampling. A sample of approximately 0.25 g was immediately placed into a 1.5 mL microcentrifuge tube and returned to −20 °C until DNA extraction. For the Dutch samples, 100–200 g of fresh kidney tissue (encompassing both the cortical and medullary regions) was homogenized using a mechanical blender. This homogenate was then stored at −20 °C. For final analysis, a 1 g aliquot of the thawed homogenate was transferred for DNA extraction.

Urine samples (ranging from approximately 4 to 15 mL), stored in 15 mL Falcon tubes, were thawed and briefly vortexed. Samples were centrifuged at 415 rcf for 5 min to obtain sediment. The supernatant was carefully discarded, and the resulting pellet was transferred to 1.5 mL microcentrifuge tube. Up to 0.25 g of sediment was used per tube for DNA isolation, adhering to the manufacturer’s protocol. The rest of the sediment was discarded.

DNA was extracted from kidney tissue and urine using the DNeasy Blood & Tissue Kit (QIAGEN, Germany) and the DNeasy PowerSoil Pro Kit (QIAGEN, Germany) respectively, following the manufacturer’s instructions. DNA was eluted in 100 μL of elution buffer for the kidney tissue samples, and in 50 μL for the urine sediment samples. The extracted DNA was then stored at −20 °C until PCR amplification. The presence of *Klossiella equi* DNA was tested by conventional PCR targeting mitochondrial multi-copy rRNA gene array (12S/16S; henceforth referred to as ID PCR) located between cytochrome c oxidase subunits 1 (*COI*) and 3 (*COIII*), using primers Api_LSUG_F and Haem_RNA_14_R (21). For a subset of 38 positive samples on the ID PCR, additional PCR reactions targeting partial mitochondrial *COI* and cytochrome b (*cytB*) were conducted. The selection of the positive samples aimed at ensuring geographic representation of all countries. PCR reaction for ID PCR was carried out in a total volume of 25 μL, containing 12.5 μL of 2x PCRBIO Taq Mix Red (PCR Biosystems, United Kingdom), 0.4 μM of each primer, and 2 μL of DNA. PCRs targeting partial *COI* and *cytB* were done in a total volume of 20 μL, containing 10 μL of Phusion Green Hot Start II High-Fidelity PCR Master Mix (Thermo Fisher Scientific, United States), 0.5 μM of each primer, and 2 μL of DNA and were run as touch-down PCRs. In all PCR assays, PCR grade water was used as negative control. To test all PCR assays two *K. equi* DNA samples obtained in previous study were used as positive control (21). Details of all PCR protocols are shown in Table 1.

**Table 1:** Primers and PCR conditions used for the identification and genetic characterization of *Klossiella equi.*

PCR	Primers	Primer sequence (5′–3′)	Annealing (°C)	Product size	Reference
ID PCR	Api_LSUG_F	TTGAGRCAGTTTGTTCCCTATCT	56	575 bp	(21)
	Haem_RNA14_R*	GAGTCGMTCAGGAAGGTTTC			
*COI*	Api_LSUF_F	GTWCGCCGGGGATAACAGGT	67–59**	1,190 bp	
	Kloss_COI_350R_rev*	CAACCGCAGCTGAATTTGGA			
*cytB*	Kloss_CytB_201F	CGTGAAATAGGAAGTGGTTGGT	67–59**	1,133 bp	
	Kloss_CytB_R	GTAAGGAAAAGGAAAGGTTAACCGC			This study

*Modified from (21). **Touch-down PCR with starting annealing temperature declining at 0.5 °C/cycle to final temperature.

PCR products were separated by electrophoresis on 2% agarose gel stained with Midori Green Advanced (Nippon Genetics Europe, Germany) and visualized under UV light. For previously selected positive samples, PCR products with the band of expected size were purified using ExoSAP-IT™ PCR Product Cleanup Reagent (ThermoFisher, United States) and sent for Sanger sequencing (Macrogen Europe, Netherlands), using the amplification primers. Obtained sequences were analyzed and aligned in Geneious Prime (Geneious Prime 2024.0.7) and compared with those available in the GenBank® database by the Basic Local Alignment Search Tool (BLAST).

### 2.3 Phylogenetic analysis

A phylogenetic tree was prepared for *COI* to assess the position of obtained sequences. The tree was constructed using a subset of unique sequences from the suborder Adeleorina available in the GenBank database. All phylogenies were inferred by IQ-TREE v.1.6.12 (32), with the best-fit evolution model selected based on the Bayesian information criterion, computed and implemented using ModelFinder (33). Branch supports were assessed by ultrafast bootstrap (UFBoot) approximation (34) and the Shimodaira-Hasegawa-like approximate likelihood ratio test (SH-aLRT) (35), both with 1,000 replications. Trees were then visualized and edited in FigTree v.1.4.4 and Inkscape v.1.3, respectively. Details are shown in figure description.

### 2.4 Statistical analysis

Statistical analyses were performed using R (version 4.4.0). PCR positivity was calculated as the proportion of PCR-positive samples among all tested samples, separately for kidney tissue and urine, as well as combined positivity defined as detection in either sample type.

Because sampling was opportunistic and no independent gold standard for intravital detection of *K. equi* is available, diagnostic performance and adjusted PCR-positive proportions were explored using a composite reference standard (CRS), defined as a positive PCR result in either kidney tissue or urine. Urine PCR performance against the CRS was evaluated by calculating sensitivity, specificity, positive predictive value (PPV) and negative predictive value (NPV), with 95% confidence intervals. Specificity and PPV may be inflated due to incorporation bias, as urine PCR contributed to the CRS definition. In addition, adjusted PCR-positive proportions were estimated using the Rogan-Gladen correction based on CRS-derived sensitivity and specificity and are reported as exploratory corrected estimates rather than inferential measures.

For paired kidney and urine samples (*n* = 82), differences in detection rates were assessed using the exact McNemar test for marginal homogeneity. Agreement beyond chance was evaluated using Cohen’s kappa (*κ*) and interpreted according to Altman (36) (poor ≤0.20, fair 0.21–0.40, moderate 0.41–0.60, good 0.61–0.80, very good 0.81–1.00).

Associations with age and sex were assessed using Fisher’s exact test in subsets of 175 horses with known age and 177 horses with known sex (mares = 89; stallions/geldings = 88). Horses were grouped into foals (<1 year), young adults (1–5 years), and mature adults (>5 years). Effect sizes were expressed as odds ratios with 95% Wilson confidence intervals. Mules and one donkey were excluded, as were 100 horses from Netherlands due to missing demographic data; in Italy, age was unavailable for two horses with recorded sex. Given uneven sampling across countries and strata, these analyses were considered exploratory. Statistical significance was defined as a two-sided *p*-value <0.05.

## 3 Results

*Klossiella equi* DNA was detected by amplification of ∼575 bp of mitochondrial DNA in 33.1% (94/284) of the equines including 31.0% (88/284) of kidney samples and 25.6% (21/82) of urine samples (*p* > 0.05). Table 2 shows the raw and Rogan-Gladen-adjusted percentages of PCR-positive samples per country, with 95% confidence intervals. Among the 82 paired samples, 26 kidneys and 21 urine samples were positive. Discordant results were observed in 17 samples, 11 kidney positive/urine negative, and 6 kidney negative/urine positive (Table 3). Cohen’s kappa coefficient was 0.5 (95% CI: 0.3–0.7), suggesting a moderate agreement.

**Table 2:** Percentage (95% confidence interval) of observed and adjusted *Klossiella equi* PCR-positive samples.

Country	Equines sampled	Kidneys (positive/total)	Urine sediment (positive/total)	Observed positivity	Adjusted positivity
Italy	46	20/46	NA	43.5 (28.9–58.9)%	66.3 (46.1–88.0)%
Romania	96	14/96	5/51	17.7 (10.7–26.8)%	27.0 (17.3–40.5)%
Spain	30	19/30	16/28	73.3 (54.1–87.7)%	100.0 (84.7–100.0)%
Czechia	12	2/12	0/3	16.7 (2.1–48.4)%	25.4 (7.2–68.3)%
Netherlands	100	33/100	NA	33.0 (23.9–43.1)%	50.3 (37.4–65.1)%
Total	284	88/284	21/82	33.1 (27.7–38.9)%	50.5 (42.5–59.1)%

**Table 3:** Comparison of the positivity/negativity across 82 paired kidney and urine samples.

	Urine positive	Urine negative
Kidney positive	15	11
Kidney negative	6	50

Using CRS, urine showed an apparent sensitivity of 65.6% (95% confidence interval [CI]: 46.8–81.4%) and apparent specificity of 100% (95% CI: 92.9–100%). Similarly, the apparent PPV was 100% (95% CI: 83.9–100%) and the NPV was 82.0% (95% CI: 70.1–90.6).

While sequences of the ID PCR were obtained for the whole subset of 38 samples, only 25 tested samples provided sequences of both *COI* and *cytB*. Specifically, we were unable to obtain *COI* sequence in three samples, *cytB* sequence in four samples and we obtained only ID PCR sequence for six samples, likely due to quality or fragmentation of the DNA The sequence analysis of this subset showed two haplotypes for *cytB* and ID PCR each and four haplotypes for *COI*. The most different haplotype for all 3 used markers was consistently detected in two samples from Italy (see Supplementary Table 1). These two isolates were the only positive non-horse samples in the entire study. Details on the haplotypes obtained and BLAST analysis are shown in Table 4. The haplotype homology differed for each marker. Regarding *COI,* the sequence identity for the three similar haplotypes ranged from 99.82 to 99.91%, while the similarity of the more distant haplotype to the rest of haplotypes was 98.27–98.37% with 15 single nucleotide polymorphisms (SNPs), one amino acid (AA) insertion and four AA substitutions. The observed sequence similarity for ID PCR and *cytB* sequences was 99.64 and 99.15% (9 SNPs and two AA substitutions), respectively.

**Table 4:** BLAST analysis results and haplotype overview.

Haplotype	Marker	No. of seq.	BLAST results (% query cover; % identity)	Accession number
Canada	ID PCR	36	MH203050 (100%; 100%)	—
Italy	ID PCR	2	MH203050 (100%; 99.64%)	PX676284
Canada	*COI*	4	MH203050 (100%; 100%)	—
Italy	*COI*	2	MH203050 (100%; 98.46%)	PX676282
Europe A	*COI*	22	MH203050 (100%; 99.91%)	PX676280
Europe B	*COI*	1	MH203050 (100%; 99.82%)	PX676281
Canada	*cytB*	26	MH203050 (100%; 100%)	—
Italy	*cytB*	2	MH203050 (100%; 99.15%)	PX676283

**Figure 2:**
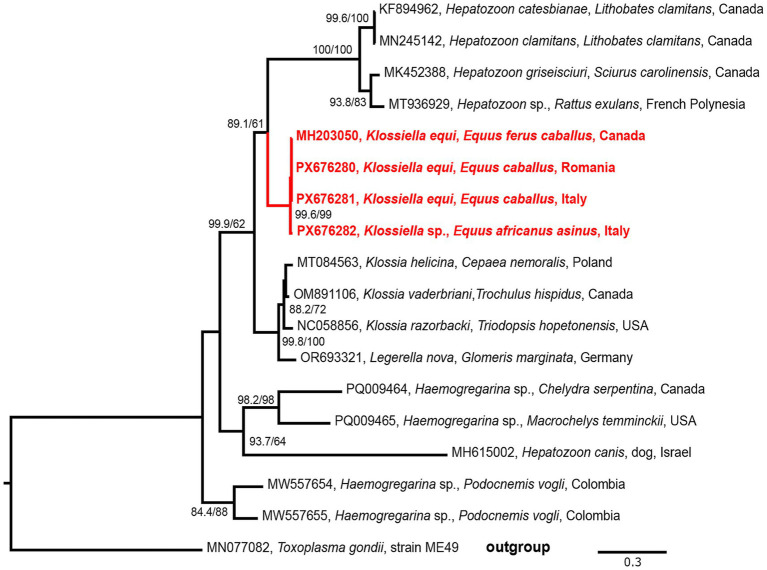
Schematic representation of maximum likelihood phylogenetic tree based on the *COI* gene sequences of available Adeleorina. The final length of the alignment was 1,491 bp and it contained 18 sequences (three originated from this study). The tree was constructed using the evolution model TPM2 + F + I + G4. One sequence of *Toxoplasma gondii* was used as an outgroup. Sequences obtained/detected in this study are marked in bold and red. The scale bar indicates the number of nucleotide substitutions per site. The bootstrap values (SH-aLRT/UFB) above the 80/95 threshold are displayed. Sequences are labeled by accession number, species, host, and country of origin (if available).

The phylogeny of *COI* placed all obtained sequences into a well-defined and highly supported clade together with the sequence of *K. equi* from Canada (21). The *COI* sequence of the Italy haplotype (PX676282) is clearly distinct from the remaining *K. equi* sequences with high branch support. Details can be found in Figure 2.

No significant difference was found between gender (*p* = 1.00). PCR positivity was 33.7% in mares (30/89) and 33.0% in stallions/geldings (29/88). Host age was significantly associated with PCR positivity (*p* < 0.001). PCR positivity was highest in young adults aged 1–5 years (56.3%; 36/64), driven largely by high rates in Italy and Spain, while foals younger than 1 year showed the lowest PCR positivity (12.5%; 1/8), including a single positive 10 month-old from Romania. Mature adults aged 6–39 years exhibited an intermediate PCR positivity (20.4%; 21/103). Detailed distributions for age and gender are summarized in Tables 5 and 6.

**Table 5:** Frequency distribution and risk analysis by gender and age.

	Category	*n*	Positive/total (%)	Odds ratio (95% CI)	*p*-value
Gender	Mares	89	30/89 (33.7%)	Ref (1.0)	1
	Stallions/geldings	88	29/88 (33.0%)	0.97 (0.52–1.81)	
Age	<1 year	8	1/8 (12.5%)	0.56 (0.07–4.79)	<0.001
	1–5 years	64	36/64 (56.3%)	5.02 (2.52–9.99)	
	>5 years (6–39 yrs)	103	21/103 (20.4%)	Ref (1.0)	

Positivity (%), Odds ratios (OR) and 95% confidence intervals (CI) are shown. Reference groups are indicated by “Ref.,” *p*-value indicates the global significance between groups within a category.

**Table 6:** Geographic and age-stratified molecular detection frequency of *K. equi* in the horse population.

Country	Age group	Total (*n*)	Positive (*n*)	Positivity (%)
Czechia	Mature adults	12	2	16.7
Italy	Foals	2	0	0
	Young adults	21	13	61.9
	Mature adults	14	4	28.6
Romania	Foals	6	1	16.7
	Young adults	13	1	7.7
	Mature adults	77	15	19.5
Spain	Young adults	30	22	73.3

Data are presented for four European countries, with horses categorized into three age groups: foals (<1 year), young adults (1–5 years), and mature adults (>5 years). Positivity represents the proportion of positive cases relative to the total sample size (*n*) in each group.

## 4 Discussion

In this study, we investigated the occurrence and genetic diversity of *K. equi* in five European countries and compared its detection in kidney tissue and urine. The data obtained demonstrate that *K. equi* occurs in equids from all investigated European countries, while genetic variation among the sequenced isolates was relatively low.

Previous data on *K. equi* distribution in Europe are sporadic. The parasite is probably distributed worldwide; however, available records involve mostly case reports. In Europe *K. equi* was first reported in Hungary (37) and later in two horses in Italy (Marcato and Benazzi, 1981). The first case in Italy was identified in one of a thousand healthy slaughtered animals, while the second one involved a horse presenting with hypoproteinemia, proteinuria, and subcutaneous edema. In both cases, the infection was associated with a mild renal inflammatory reaction. Apart from Hungary, where *K. equi* was described (37), there are no other data coming from Europe. While Sasmore (38), Hartman (39), Vetterling and Thompson (24) and Reppas and Collins (40) examined for *K. equi* series of ~20–60 individuals, most other findings were incidental infections reported in a single animal (26, 22, 23, 25, 27, 29, 48, 52–54).

As a majority of available data originates from postmortem examinations, one of our objectives was to evaluate the usefulness of DNA detection in urine as a reliable intravital diagnostic tool. Presence of sporocysts in urine has been repeatedly demonstrated and represents an apparent way how parasites leave the infected host (25, 26, 40, 55), however, comparative data on presence/absence in kidneys and urine are missing. Our analysis of the 82 paired kidney-urine samples demonstrated moderate agreement between the two methods (Cohen’s kappa = 0.5), indicating only partial concordance between sample types. This suggests variability in PCR detectability across matrices rather than consistent agreement. The observed discordance may reflect a combination of biological and methodological factors, including intermittent shedding, low parasite burden, uneven renal distribution of infection, and potential PCR inhibition. Acquired immunity may also contribute to reduced parasite replication and shedding, as described in coccidial infections where prior exposure can limit parasite multiplication (41). However, the relative contribution of these mechanisms cannot be distinguished in the present study. Collectively, these factors likely account for the moderate agreement observed between kidney and urine PCR results, which highlights the utility for non-invasive field diagnostics. In clinical practice, this could provide a practical and cost-effective tool for live animal screening and long-term surveillance.

Despite moderate sensitivity, urine PCR showed apparent high specificity when evaluated against the composite reference standard. Interpretation of diagnostic performance should be made with caution due to the use of a composite reference standard defining positivity as PCR detection in either kidney or urine. Because urine PCR contributed to this definition, estimates of specificity and positive predictive value may be inflated and are not independent of the reference standard due to incorporation bias. Therefore, the observed specificity and PPV, including values reaching 100%, should not be interpreted as true measures of diagnostic accuracy. Urine PCR may nonetheless be useful as a non-invasive screening or surveillance tool for *K. equi*, pending validation against an independent reference standard. Likewise, Rogan-Gladen-adjusted positivity estimates should be considered exploratory, as sensitivity and specificity were derived from the same composite framework.

The analysis of concatenated mitochondrial markers revealed four distinct haplotypes within the study population, indicating overall low genetic diversity in the available data set. Three haplotypes differed from the dominant lineage by only one or two synonymous substitutions within the *COI* gene, indicating close genetic relatedness among these variants. A putative predominant “European haplotype” was identified across all sampled countries, while the remaining minor variants were identified exclusively from Italian samples. Comparison with existing data showed that four of our samples were identical to the Canadian sequences described by Léveillé et al. (21), while the majority of sequenced samples (*n* = 22) differed only by one SNP in *COI*.

The greatest genetic divergence was observed in two divergent samples associated with non-horse equids: one isolated from a donkey (Avellino; Campania region—southern Italy) and the other from a mule (Frosinone; Lazio region—central Italy). To our knowledge, this represents the first molecular detection of *Klossiella* in a mule. These two isolates originated from the only PCR-positive non-horse equids in our dataset and exhibited marked divergence across all three mitochondrial markers. Most notably, the *COI* gene showed an approximate 1.5% divergence from the predominant lineage with five amino acid substitutions, a pattern also supported by the *COI* phylogenetic analysis. However, whether these isolates represent distinct lineages or fall within the random intraspecific variability requires more research.

The Adeleorina includes 10 recognized genera and likely tens or even hundreds of individual species. However, mitochondrial sequences are only available from nine species. Incomplete sampling of related species restricts resolution of both interspecific and intraspecific diversity. Although the observed divergence in *COI* and *cytB* suggests genetic differentiation, it is insufficient to support species delineation of Italian lineages. Amplification of all three mitochondrial markers was not successful for all positive samples, likely due to low template concentration or DNA fragmentation, meaning that additional diversity may remain undetected. Future studies employing more sensitive approaches such as nested PCR or next-generation sequencing, as well as quantitative PCR to estimate parasite burden, would help clarify phylo-geographic structure and potential host associations (42).

Our study indicates occurrence of *K. equi* across European equid populations, consistent with recent findings from North America (31). Yet the clinical significance of the *K. equi* remains uncertain. Due to the opportunistic nature of the sampling, detailed clinical data was not consistently available. Previous work (21) described renal infection without detectable clinical abnormalities, suggesting subclinical course. Only two recent reports linked *K. equi* infection with clinical renal disease (25, 26). Similarly, *Klossiella muris* has been reported to have relatively high prevalence in wild rodents without overt renal dysfunction (43), although histological changes may occur in association with higher infection intensity. Few clinically significant renal pathogens are recognized in horses. The presumably asymptomatic nature of *K. equi* infection stands in sharp contrast to leptospirosis. Although both pathogens may be shared in urine, their clinical consequences differ markedly with the latter causing severe systemic and renal disease (44–46).

Several limitations should be considered when interpreting *K. equi* results. The sampling was opportunistic and based mainly on slaughterhouse and post-mortem material; therefore, results are not interpreted as true population-level prevalence estimates, but rather as molecular detection frequencies in the sampled equids. The dataset was unevenly structured across countries and host species, with different sample sizes and a strong predominance of horses over mules and a single donkey, limiting robust species- and country-level comparisons. The Spanish cohort had a distinct management history, as animals of different origins raised in northern regions were later transported to a southern feedlot where high stocking densities contributing to locally intense transmission, introducing potential bias in interpreting higher PCR positivity in Spain. In addition, age-related patterns should be interpreted cautiously due to uneven age distributions between countries. Also, a methodological limitation is that kidney processing differed between countries, with Dutch samples derived from larger homogenized tissue volumes (100–200 g) compared to approximately 0.25 g elsewhere, which may have affected detection sensitivity and should be considered when interpreting cross-country comparisons.

## 5 Conclusion

The fact that *K. equi* has been rarely diagnosed in Europe should not be interpreted as evidence of negligible importance. Documented occurrence across all five European countries examined suggests broad distribution and detection of *K. equi* DNA or sporocysts may be relevant in the differential context of renal investigations. Detection of this intracellular parasite deserves attention of clinicians, especially in cases of clinical nephropathies.

## Data Availability

The original contributions presented in the study are publicly available. This data can be found at the National Center for Biotechnology Information (NCBI) using accession numbers PX676280–PX676284.
